# Editor’s Outlook

**Published:** 1893-07

**Authors:** 


### EDITOR'S OUTLOOK.

### MAN'S INDISCRETIONS.

The span of human life is shortened sometimes by the disorders of nature, but mainly by our own ignorance and thoughtlessness. We are cut off, though not suddenly yet prematurely, by disease, inappropriate labor, improper, or insufficient, or immoderate foods, and a thousand other irregularities, which would have no existence after a few generations of general enlightenment.

That man, like nature herself, has an inclination towards a certain methodism in his goings out and comings in, cannot be denied, since he is one of the children of nature; but the tendency is frequently shown in so irrational a manner as to neutralize its advantage. He takes his meals at a regular hour—that is his instinct; but he appoints that hour, not according to the dictates of nature, but fashion.

He goes to bed once in twenty-four hours, but the time depends upon circumstances, although these, generally speaking, are completely under his own control. He eats and drinks not only to satisfy hunger and thirst, but gluttony and an inclination for unwholesome stimulants. When this mode of living meets its due reward, and he becomes unwell, all these irregularities are amended under the directions of the physician. He is reduced to order; he falls into the general, not pedantic methodism of nature; and he gets well? Not exactly. He gets well enough to begin his course anew, as if nothing had happened; but the mischief is done—he has cribbed a certain space from his allotted span. There is nothing more absurd and meaningless than that expression—getting well. We *never* get well. Every bygone disease has, in the common phrase, driven a nail in our coffin. The very act of repairing is an added injury; every dose of medicine contains some drops of poison, which even if the disease is cured, subject the patient to a longer or shorter period of convalescence.

A most advantageous custom and one which promotes health to the body and brain, is that of citizens spending the hottest weeks of the year in the country; there cannot be a doubt of its revivifying and regenerating effects, when the time is occupied in a proper manner and the hablts of eating, drinking and exercise are dictated by a judicious reference to the ascertained laws of our being. A summering in the country will be beneficial to the body, in proportion as the whole time of daylight, from early breakfast until sundown is spent in active pleasurable exercise in the open air; exercise which, as often as taken, should be to the extent of some little fatigue. As to young men and old the best plan is to be afoot from morning until night, in fishing, hunting wild animals, religiously sparing the sweet birds of the wood, whose gleeful songs, as if in welcome of our arrival ought to smite any generous heart with reproaches, for even the thought of murdering them in cold blood.

As to women and girls, especially those who are burdened with family cases at home, or are weighed down with that greater load, fashionable life, the better plan is to hie to the seashore, mountains or by the lake as soon as the weather is permissible. Keep away from the fashion circles; indulge freely in exercise out doors; row a boat or ride a horse; walk by the earliest dawn, or frolic by the clear moonlight of summer; wear loose clothing and all the while eating not an atom except at the three regular meals of the day; getting all the sleep possible but only during the hours of darkness. Acting thus, few will fail of real and lasting renovation, by spending a summer in the country.

---

Two months remain for our readers to avail themselves of our Encyclopedia offer. We venture to say that no periodical has ever offered such a valuable premium as this and it is our desire that no one be disappointed in not procuring the treasure.

In order to answer the many inquiries that have come to us asking a description of the Encyclopedia, we take the liberty of giving our readers a few *voluntary* words of praise for the Journal and Encyclopedia, from people already among our readers and who have accepted our offer:

Lowell, Mass., June 1.

To Publishers Hall's Journal of Health.

Journal and premium have reached me in good order. I am very well pleased with the Encyclopedia. It fulfills all that is claimed for it and each of the thirty volumes are neatly and conveniently turned out. Yours, JOHN J. LYONS.

Mrs. H. E. Tyler, of Fairbury, Ill., writes:

I have received Chambers Encyclopedia, offered as a premium by you with the Journal and am very well pleased with it and think it will be a convenient size for reference. Many thanks.

Miss C. E. Marshall, a lady of culture and refinement, who makes her home at the Barrett House, New York City, during the winter and travels during the summer months, enclosed us a check for a renewal of three subscriptions to the Journal, just before she sailed for Italy recently with these words: "I wish to say that your Journal has given me so much pleasure and I have learned much from its valuable hints. Wish you every success."

These are sample letters of what come to our offices regarding our magazine.

Our Recreation Bureau will be continued in the issues of August and September for the benefit of those who desire to take advantage of its unbounded facilities for learning where to spend a vacation or how to go and where to stop in Chicago on your World's Fair visit.

Please bear in mind all information is furnished free and circulars or pamphlets will be forwarded to you upon application.

